# Anthropometric measurements as a potential non-invasive alternative for the diagnosis of metabolic syndrome in adolescents

**DOI:** 10.20945/2359-3997000000100

**Published:** 2019-02-01

**Authors:** Silmara Salete de Barros Silva Mastroeni, Marco Fabio Mastroeni, John Paul Ekwaru, Solmaz Setayeshgar, Paul J. Veugelers, Muryel de Carvalho Gonçalves, Patrícia Helen de Carvalho Rondó

**Affiliations:** 1 Universidade da Região de Joinville Universidade da Região de Joinville Departamento de Educação Física Joinville SC Brasil Departamento de Educação Física, Universidade da Região de Joinville (Univille), Joinville, SC, Brasil; 2 University of Alberta University of Alberta School of Public Health Population Health Intervention Research Unit Edmonton Alberta Canada Population Health Intervention Research Unit, School of Public Health, University of Alberta, Edmonton, Alberta, Canada; 3 Universidade da Região de Joinville Universidade da Região de Joinville Joinville SC Brasil Programa de Pós-Graduação em Saúde e Meio Ambiente, Universidade da Região de Joinville (Univille), Joinville, SC, Brasil; 4 Universidade da Região de Joinville Universidade da Região de Joinville Departamento de Ciências Biológicas Joinville SC Brasil Departamento de Ciências Biológicas, Universidade da Região de Joinville (Univille), Joinville, SC, Brasil; 5 Universidade de São Paulo Universidade de São Paulo Faculdade de Saúde Pública Departamento de Nutrição São Paulo SP Brasil Departamento de Nutrição, Faculdade de Saúde Pública, Universidade de São Paulo (FSP-USP), São Paulo, SP, Brasil

**Keywords:** Adolescents, metabolic syndrome, anthropometric measurements, neck circumference, ROC curve

## Abstract

**Objective:**

To identify which anthropometric measurement would be the best predictor of metabolic syndrome (MetS) in Brazilian adolescents.

**Subjects and methods:**

Cross-sectional study conducted on 222 adolescents (15-17 years) from a city in southern Brazil. Anthropometric, physical activity, blood pressure and biochemical parameters were investigated. MetS criteria were transformed into a continuous variable (MetS score). Linear regression analyses were performed to assess the associations of BMI, hip circumference, neck circumference (NC), triceps skinfold, subscapular skinfold and body fat percentage with MetS score. ROC curves were constructed to determine the cutoff for each anthropometric measurement.

**Results:**

The prevalence of MetS was 7.2%. Each anthropometric measurement was significantly (p < 0.001) associated with MetS score. After adjusting for potential confounding variables (age, sex, physical activity, and maternal education), the standardized coefficients of NC and body fat percentage appeared to have the strongest association (beta = 0.69 standard deviation) with MetS score. The regression of BMI provided the best model fit (adjusted R^2^ = 0.31). BMI predicted MetS with high sensitivity (100.0%) and specificity (86.4%).

**Conclusions:**

Our results suggest that BMI and NC are effective screening tools for MetS in adolescents. The early diagnosis of MetS combined with targeted lifestyle interventions in adolescence may help reduce the burden of cardiovascular diseases and diabetes in adulthood.

## INTRODUCTION

The prevalence of overweight and of its comorbidities have increased in adolescents and have reached epidemic proportions in both developed and developing countries (1). The increase in the prevalence of overweight is higher in developing than in developed countries, with reported increases of 65% and 48% between 1990 and 2010, respectively (1). In 2015, 23.7% of 13-17-year-old Brazilian adolescents were overweight (including obese) and 7.8% were obese (2). The rise in the prevalence of childhood overweight will result in significant health problems and financial burdens in the future, therefore warranting comprehensive prevention efforts (3).

One of the consequences of being obese or overweight is the risk of developing metabolic syndrome (MetS) (4), a complex condition of multiple, interrelated risk factors for cardiovascular diseases (CVDs) and diabetes (5). These risk factors include elevated fasting glucose and triglyceride levels, high blood pressure, low high-density lipoprotein cholesterol (HDL-c) levels, and central adiposity (5). According to the International Diabetes Federation (IDF), MetS is defined as the presence of elevated waist circumference plus two of the following four criteria: high blood pressure, elevated triglyceride and fasting plasma glucose levels, and decreased HDL-c levels (6).

The prevalence of MetS is increasing worldwide due to the rise in obesity and poor lifestyle (5). Excess body weight is the primary cause of MetS due to the increase in insulin production, as well as the likelihood of developing insulin resistance, a central pathophysiological factor in the development of MetS (7,8). Insulin resistance has multiple metabolic effects in the organism, including the increased synthesis of very-low-density lipoprotein cholesterol and cholesterol, resistance to the action of insulin on lipoprotein lipase in peripheral tissues, degradation of HDL-c, enhanced sympathetic activity, and increased formation of plaque which is associated with high blood pressure (8,9). Another important point regarding the adipose tissue is the production of leptin, adiponectin, and resistin, as well as interleukin-6, tumor necrosis factor-alpha and plasminogen activator inhibitor-1 (8,9). All these cytokines are involved in the inflammatory process (8,9), indicating that the pathological consequences of excess body fat involve different tissues and organs (8,9).

In the last decade, MetS has been identified in younger populations (4,10–12). This is particularly concerning given the potential earlier manifestations of MetS outcomes, such as type 2 diabetes and CVDs (4,13). The early identification of MetS would permit early preventive actions (14) designed to reduce the burden of type 2 diabetes and CVDs later in life (15). However, there is no universal or uniform definition of MetS in younger populations (12). Since the prevalence of MetS in children and adolescents shows significant disparities among studies and the use of multiple logistic regression analysis provided controversial results, some authors (12) suggested the use of a continuous variable of MetS (MetS score) to overcome these limitations (12).

In recent years, several authors have suggested the use of neck circumference (NC) to identify MetS in adult populations (16–24). Other studies proposed the use of NC (25–27), BMI (15,25,28–30), waist circumference (11,12,15,26,29), waist-hip ratio (25), waist-height ratio (29), wrist circumference (26), skinfold thickness (31), conicity index (29), and fat and lean body mass index (BMI) (30) to predict MetS in adolescents. More recently, a national study assessed the prevalence of MetS and its components in a large sample of Brazilian adolescents (The ERICA Study) (11). Another national study the authors evaluated the validity of continuous metabolic syndrome score for predicting MetS and to determine the cutoff values in a representative sample of Iranian children and adolescents (The CASPIAN-V Study) (12). However, to the best of our knowledge, no study simultaneously compared six different anthropometric measurements (BMI, hip circumference, NC, triceps skinfold, subscapular skinfold and body fat percentage (BF%)) in the same population for the identification of MetS in Brazilian healthy adolescents.

Since the diagnosis of MetS is invasive, expensive, and labor intensive on the part of health professionals, noninvasive and low-cost methods are needed, particularly in low-resource settings. The aim of this study was to evaluate BMI, hip circumference, NC, triceps skinfold, subscapular skinfold and BF% as potential alternatives for the diagnosis of MetS in adolescents.

## SUBJECTS AND METHODS

### Subjects and study design

This was a cross-sectional study conducted in two phases on 15- to 17-year-old high school students from Joinville, a city of about 500,000 inhabitants, Santa Catarina, Brazil. Details of the recruitment process have been described previously (32). In the first phase, 2,195 students completed a short survey on socioeconomic and demographic characteristics. An informed consent form was handed out to obtain agreement for participation from their parents/guardians and 1,104 (50.3%) students returned the signed consent form (32).

All of the 1,104 students were invited to participate in the second phase. They were contacted by phone and by a personal visit in the residence and were informed about the day and place of data collection. At the end of the study, 222 students participated in the data collection (32). Assessments in this phase included anthropometric measurements (weight, height, waist and hip circumferences, NC, triceps and subscapular skinfold thickness, and BF%), physical activity, biochemical analyses, and blood pressure measurement. Blood samples were drawn from each participant to assess the levels of fasting insulin, fasting glucose, total cholesterol, low-density lipoprotein cholesterol (LDL-c), HDL-c, and triglycerides.

The study was carried out in accordance with the Declaration of Helsinki and the Research Ethics Committee of the University of Joinville Region approved this study (Approval No. 005/2007).

### Data collection

The anthropometric measurements were made in the morning after an overnight fast, with the subjects wearing light clothing and no shoes. The adolescents were weighed on a Filizola^®^ digital scale (Curitiba, PR, Brazil; capacity of 180 kg) to the nearest 0.1 kg. Height was measured with a Cardiomed^®^ stadiometer (Curitiba, PR, Brazil; 200 cm) to the nearest 0.1 cm. Waist circumference was measured midway between the lowest rib and the top of the iliac crest during the mid-expiratory phase. Hip circumference was measured with the tape at the widest portion of the buttocks. Neck circumference was measured horizontally above the cricothyroid cartilage with the tape not compressing the skin (33). All circumferences were measured with a flexible tape (Cardiomed^®^; 150 cm) to the nearest 0.1 cm.

A Cescorf^®^ skinfold caliper (Porto Alegre, RS, Brazil) was used to measure triceps and subscapular skinfold thickness at a pressure of 10 g/mm^2^ over the contact surface area to the nearest 0.1 mm. Triceps skinfold thickness was measured on the back of the arm and at a point midway between the acromion and olecranon process. With the arm hanging loosely, subscapular skinfold thickness was measured 2 cm below the inferior angle of the right scapula. All anthropometric variables were measured three times and the arithmetic mean of these measurements was used as the final result.

Body mass was evaluated by calculating the BMI [weight (kg)/height (m^2^)] following the classification of the World Health Organization for the calculation of BMI according to age and sex (34). Body fat percentage was obtained by foot-to-foot bioelectrical impedance analysis using a BIA 310e^®^ Bioimpedance Analyzer (Biodynamics Corporation, Shoreline, WA, USA).

The diastolic (DBP) and systolic blood pressure (SBP) was measured using the HDI/Pulse Wave^TM^ CR-2000 Research Cardiovascular Profiling System (Hypertension Diagnostic, Inc., Eagan, MN, USA), with the adolescent lying on a gurney after a 10-min resting period.

Information about maternal education and physical activity was collected by interview. Physical activity was classified using the International Physical Activity Questionnaire (IPAQ) (35).

### Biochemical analysis

Approximately 15 ml of venous blood was drawn from the antecubital vein of each subject. All blood samples were collected in the morning after an overnight fast. Within 30 min, the remaining blood serum was separated by centrifugation at 3,500 rpm for 10 min at 4 °C, immediately aliquoted, and frozen at −70 °C until the time of analysis.

Fasting glucose, LDL-c, and HDL-c were analyzed by colorimetric enzymatic methods on the Bayer ADVIA 1650 automated analyzer using the GLUO, D-LDL and D-HDL kits, respectively (Siemens Diagnostics^®^, Tarrytown, NY, USA). Total cholesterol and triglycerides were measured with the Bayer ADVIA Centaur automated analyzer using the Cholesterol and Triglycerides Liquiform kits, respectively (Labtest Diagnostica^®^, Vista Alegre, MG, Brazil). Insulin was assayed by a chemoluminescence method on the Bayer ADVIA Centaur automated analyzer with IRI Bayer ADVIA kit, analytical sensitivity of 0.5 μIU/ml (Siemens Diagnostics^®^). The homeostatic model assessment for insulin resistance (HOMA-IR) index was calculated using the equation [HOMA-IR = fasting insulin [(μIU/ml) × fasting glucose (mmol/l)/22.5]. All measurements were performed in a laboratory accredited by the Brazilian Society of Clinical Analysis.

### Definition of metabolic syndrome and metabolic syndrome score

MetS was defined using age- and sex-specific cutoff points for each component, according to Jollife and Janssen (6). Each MetS component growth curve was linked to the corresponding Adult Treatment Panel and the IDF cut point (6) In the present study, MetS was defined according to the IDF criteria as increased waist circumference and two of the following four criteria: elevated blood pressure, triglyceride and fasting plasma glucose or decreased HDL-c (6).

For the purpose of the present study, a MetS score was created using the sex- and age- specific Z-score cutoff points for the following variables: waist circumference, SBP, DBP, triglycerides, HDL-c, and HOMA-IR according to Eisenmann (36). The MetS score was chosen due to the lack of a standard definition of MetS for children or adolescents and because its prevalence in the population is still low (36). Since HDL-c concentration is inversely related to metabolic risk, the values of this variable were multiplied by −1. A higher score indicates a less favorable MetS profile (36).

### Statistical analysis

The Statistical Package for the Social Sciences (SPSS), version 22.0, was used for statistical analysis. Central tendency and absolute and relative frequencies were estimated as descriptive statistics. Continuous variables are reported as median and interquartile range. The chi-square test was used to compare the prevalence of categorical variables according to the presence of MetS. The Mann-Whitney *U* test was applied to compare the medians of general characteristics according to the presence of MetS.

Spearman correlation coefficients were calculated to evaluate the association of the anthropometric parameters with MetS score, MetS components and HOMA-IR. Multiple linear regression analysis was applied to analyze the relationship between the anthropometric parameters and MetS score. Since the anthropometric parameters showed skewed distributions, they were log-transformed. Multivariable linear regression models were developed adjusting for age, sex, physical activity and maternal education. The standardized regression coefficients from these models were used to compare the relative effects of the anthropometric measurements on MetS score, regardless of the anthropometric measurement units.

We also carried out analyses to determine cutoff values of BMI, hip circumference, NC, triceps and subscapular skinfolds, and BF% to predict MetS. These cutoffs were determined by constructing receiver operating characteristic (ROC) curves for girls and boys separately. The area under the curve (AUC) was used as a measure of the diagnostic power of the test, considering the anthropometric measurements investigated. The greater the AUC, the greater the discriminatory power of the anthropometric measurement. Subsequently, the sensitivity (proportion of individuals with a diagnosis of MetS who were identified as having MetS by the anthropometric measure) and specificity (proportion of individuals without MetS who were identified as not having MetS by the anthropometric measure) were determined. The outcome, MetS score, was analyzed separately for each anthropometric parameter (BMI, hip circumference, NC, triceps and subscapular skinfolds, and BF%). Statistical significance was defined as p < 0.05.

## RESULTS

All analyses were conducted in the second phase of the study (n = 222), corresponding to individuals who returned the consent form of the first phase (n = 1,104). The Mann-Whitney *U* test showed no significant difference in maternal education or BMI (p = 0.204 and p = 0.252, respectively) between adolescents enrolled in the first and second phases. Excess body weight (overweight and obesity) was observed in 20.3% of the participants and 7.2% had MetS. Table 1 shows the general characteristics of the adolescents according to the presence of MetS. All anthropometric and metabolic parameters were significantly (p < 0.05) higher in adolescents with MetS, except for HDL-c. No significant differences in age, sex, maternal education or physical activity were observed between adolescents with and without MetS (Table 1).

**Table 1:** Socio-demographic and biochemical characteristics of Brazilian adolescents according to the presence of metabolic syndrome

Variable	Metabolic syndrome* (n = 222)		p
	Absent (n = 206)	Present (n = 16)	
Age (years)	16.0 (1.0)	16.0 (1.0)	0.699†
Girls (%)	125 (60.7%)	10 (62.5%)	0.889‡
Maternal education (years)	8.0 (6.0)	7.0 (7.0)	0.302†
Physical activity (min/week)	2,160.0 (3,441.5)	2,641.0 (3,771.8)	0.647†
BMI (kg/m^2^)	21.0 (3.6)	30.2 (8.3)	< 0.001†
Waist circumference (cm)	69.7 (8.7)	93.6 (18.3)	< 0.001†
Hip circumference (cm)	94.6 (8.2)	112.0 (15.0)	< 0.001†
Neck circumference (cm)	32.5 (5.0)	36.1 (5.7)	< 0.001†
Triceps skinfold (mm)	13.1 (10.1)	30.2 (9.2)	< 0.001†
Subscapular skinfold (mm)	11.3 (6.7)	33.7 (15.8)	< 0.001†
Body fat percentage (%)	18.0 (12.7)	27.7 (5.9)	< 0.001†
Systolic blood pressure (mmHg)	116.0 (13.0)	124.5 (14.0)	0.002
Diastolic blood pressure (mmHg)	61.0 (8.0)	66.0 (13.0)	0.001
HDL-c (mg/dL)	62.0 (15.3)	54.5 (17.5)	0.080
Triglycerides (mg/dl)	82.0 (40.3)	107.0 (62.3)	0.011
Fasting glucose (mg/dl)	102.0 (13.0)	106.5 (9.3)	0.008
Insulin (mIU/l)	9.1 (6.8)	16.6 (18.0)	< 0.001†
HOMA-IR	2.3 (1.8)	4.5 (5.5)	< 0.001†

BMI: body mass index; HDL-c: high-density lipoprotein cholesterol; HOMA-IR: homeostasis model assessment-insulin resistance.

* Values were expressed as the median (interquartile range).

† Mann-Whitney U test.

‡ Chi-square test.

The Spearman correlation coefficients of all anthropometric parameters were positively correlated (p < 0.001) with MetS score and HOMA-IR. The correlation coefficients of MetS score (rho = 0.46), triglycerides (rho = 0.21), waist circumference (rho = 0.81) and HOMA-IR (rho = 0.39) with BMI were higher when compared to the other anthropometric parameters (hip circumference, NC, triceps and subscapular skinfolds, and BF%). However, SBP (rho = 0.41) and HDL-c (rho = −0.39) showed a higher correlation with NC than with BMI, hip circumference, triceps and subscapular skinfolds, or BF%.

The results of linear regression analysis are summarized in Table 2. All anthropometric variables were significantly (p < 0.001) associated with MetS score in both unadjusted and adjusted analysis. After adjusting each model for potential confounding variables (age, sex, physical activity, and maternal education), the standardized coefficients of log transformed values of NC and BF% indicated similar associations with MetS score. For each standard deviation (SD) increase in log NC or in log BF%, the MetS score increased by 0.69 SD. The proportion of variance explained (adjusted R^2^) was higher for the regression analysis that included BMI when compared to any of the other anthropometric measurements (Table 2), i.e., BMI, age, sex, physical activity, and maternal education together explained 31% of the variation in MetS score.

**Table 2:** Association between log transformed values of the anthropometric measurements and metabolic syndrome score among Brazilian adolescents

		Standardized coefficient*	SE	95% CI	*p*	Adjusted R^2^†
**Unadjusted analysis**						
	BMI	0.57	0.06	0.45-0.69	< 0.001	0.32
	Hip circumference	0.52	0.06	0.40-0.64	< 0.001	0.27
	Neck circumference	0.35	0.06	0.23-0.47	< 0.001	0.12
	Triceps skinfold	0.40	0.06	0.28-0.52	< 0.001	0.15
	Subscapular skinfold	0.51	0.06	0.39-0.63	< 0.001	0.25
	Body fat percentage	0.33	0.07	0.18-0.46	< 0.001	0.10
**Adjusted analysis**‡						
	BMI	0.57	0.06	0.45-0.69	< 0.001	0.31
	Hip circumference	0.51	0.06	0.39-0.63	< 0.001	0.25
	Neck circumference	0.69	0.09	0.51-0.87	< 0.001	0.22
	Triceps skinfold	0.56	0.08	0.40-0.72	< 0.001	0.21
	Subscapular skinfold	0.53	0.06	0.41-0.65	< 0.001	0.26
	Body fat percentage	0.69	0.09	0.51-0.87	< 0.001	0.21

BMI: body mass index; SE: standard error of the mean; CI: confidence interval.

* Standardized regression coefficients to compare the relative effects of the anthropometric measurements on MetS score, regardless of the anthropometric measurement unit.

† Adjusted R^2^: proportion of variance explained including anthropometric measurement and confounders (age, sex, physical activity, and maternal education) as independent variables, and MetS score as the dependent variable.

‡ Each model was adjusted for age, sex, physical activity, and maternal education.

**Figure 1:**
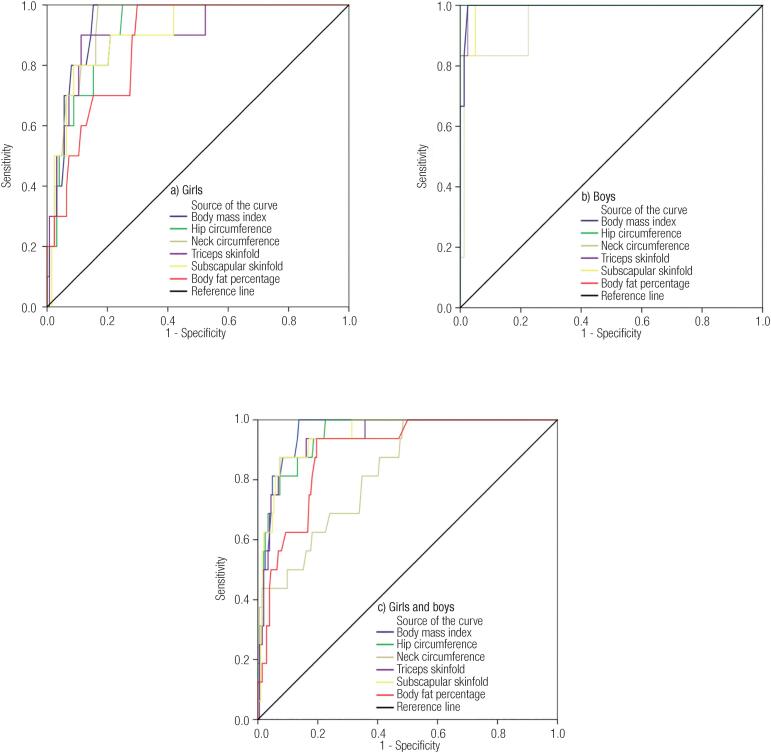
Receiver operating characteristics (ROC) curves for the prediction of metabolic syndrome using anthropometric measurements in adolescents. a) Girls. b) Boys. c) Girls and boys.

Table 3 and Figure 1 show the AUC for the anthropometric variables associated with metabolic syndrome according to sex. Higher AUC values were observed for boys (95.4% to 100.0%) when compared to girls (87.4% to 94.1%) and all differences in AUC were statistically significant (p < 0.001). Hip circumference and BMI showed the highest AUC for boys and girls, respectively.

**Table 3:** Area under the curve of the anthropometric measurements in the assessment of metabolic syndrome among Brazilian adolescents

	Boys (n = 87)	Girls (n = 135)	Both (n = 222)
Variable	AUC (95% CI)	AUC (95% CI)	AUC (95% CI)
BMI (kg/m^2^)	99.5 (98.3-100.0)	94.1 (89.8-98.4)	96.2 (93.5-98.9)
Hip circumference (cm)	100.0 (100.0-100.0)	91.3 (85.2-97.5)	95.0 (91.2-98.7)
Neck circumference (cm)	95.4 (88.4-100.0)	93.8 (89.1-98.5)	82.8 (73.6-92.0)
Triceps skinfold (mm)	99.6 (98.5-100.0)	90.4 (80.8-100.0)	94.3 (89.6-99.0)
Subscapular skinfold (mm)	99.2 (97.3-100.0)	90.5 (82.3-98.7)	94.8 (90.5-99.1)
Body fat percentage (%)	99.2 (97.3-100.0)	87.4 (79.4-95.4)	89.2 (82.7-95.7)

BMI: body mass index; AUC: area under the curve; CI: confidence interval.

The optimal cutoffs, sensitivity and specificity of the anthropometric variables related to increased MetS risk are shown in Table 4. Except for triceps skinfold and BF%, the other cutoffs were higher in boys than in girls. High sensitivity (100.0%) and specificity (> 70.0%) were found for almost all anthropometric measurements in both sexes. Hip circumference (100.0% sensitivity and specificity) and BMI (100.0% sensitivity and 84.8% specificity) were the best predictors of MetS in boys and girls, respectively. BMI was also the best predictor of MetS when boys and girls were combined (100.0% sensitivity and 86.4% specificity).

**Table 4:** Optimal cutoff, sensitivity and specificity of the anthropometric measurements for metabolic syndrome among Brazilian adolescents according to sex

Variable	Boys (n = 87)			Girls (n = 135)			Both (n = 222)		
	Cutoff	Sensitivity (95% CI)	Specificity (95% CI)	Cutoff	Sensitivity (95% CI)	Specificity (95% CI)	Cutoff	Sensitivity (95% CI)	Specificity (95% CI)
BMI (kg/m^2^)	27.2	100.0 (61.0-100.0)	97.5 (91.4-99.3)	24.0	100.0 (72.3-100.0)	84.8 (77.5-90.0)	24.1	100.0 (80.6-100.0)	86.4 (81.1-90.4)
HC (cm)	106.9	100.0 (61.0-100.0)	100.0 (95.5-100.0)	99.4	100.0 (72.3-100.0)	75.0 (67.0-82.0)	99.5	100.0 (80.6-100.0)	77.7 (71.5-82.8)
NC (cm)	36.4	100.0 (61.0-100.0)	76.5 (66.3-84.4)	32.7	100.0 (72.3-100.0)	83.1 (75.5-88.7)	32.7	100.0 (80.6-100.0)	51.5 (44.4-58.0)
TS (mm)	20.8	100.0 (61.0-100.0)	97.5 (91.4-99.3)	25.0	90.0 (59.6-98.2)	88.7 (82.1-93.2)	21.0	93.8 (71.7-98.9)	83.8 (78.4-88.4)
SS (mm)	22.9	100.0 (61.0-100.0)	95.0 (88.0-98.1)	18.2	90.0 (59.6-98.2)	79.0 (71.3-85.4)	18.2	93.8 (71.7-98.9)	82.8 (77.3-87.5)
BF (%)	18.0	100.0 (61.0-100.0)	95.0 (88.0-98.1)	24.2	100.0 (72.3-100.0)	70.2 (63.6-79.1)	23.6	100.0 (80.6-100.0)	80.4 (74.5-85.3)

BMI: body mass index; HC: hip circumference; NC: neck circumference; TS: triceps skinfold; SS: subscapular skinfold; BF: body fat percentage; CI: confidence interval.

## DISCUSSION

In this study, we observed that BMI, hip circumference, NC, triceps skinfold, subscapular skinfold and BF% were associated with MetS score. Although BMI was the strongest predictor of MetS, all other anthropometric measurements showed good or high sensitivity and specificity to identify MetS in boys and girls. Additionally, for screening purposes and clinical practice, we tried to determine the cutoff values of the six anthropometric measurements related to increased MetS risk. Our results suggest that BMI and NC are effective screening tools for MetS in adolescents.

The prevalence of MetS observed in this study (7.2%) was higher than that reported in other studies involving Brazilian adolescents (2.6%) (11), Iranian children and adolescents (5.0%) (12), Algerian adolescents (0-4.0%) (25), Greek adolescents (3.0%) (37), young Thai adults (5.9%) (38), and Korean adolescents (2.0%) (39). On the other hand, the prevalence was lower than that reported for adolescents in Brazil (12.8%) (14), and in Puerto Rico (16.8%) (40). Our study confirmed previous evidence (4,11) of a higher prevalence of MetS in overweight (33.0%) compared to normal weight adolescents (0.6%). The worldwide prevalence of MetS among adolescents is on average 10.0%, ranging from 2.0% among normal weight adolescents to 32.0% among obese individuals (4). The wide variability in MetS prevalence among studies is partly due to the use of different criteria to define MetS. The use of different criteria to identify MetS and the lack of a universal definition for adolescents make it difficult to interpret and compare the results (41,42).

Few studies conducted on adolescents (38,41) have associated body composition, measured by dual-energy X-ray absorptiometry (DXA), with MetS. Some authors have indicated BMI as the best anthropometric parameter to detect MetS in children and adolescents (15,30), in agreement with our results. We also showed that NC and BF% are strong predictors of MetS, corroborating the results of other authors (26). Although NC has been suggested to identify overweight in children (43–45) and adolescents (45,46) or to predict CVDs and MetS in the adult population (16–21,24), this parameter was recently investigated as an anthropometric parameter to predict MetS in apparently healthy adolescents (22,26,27). Since NC is a low-cost, time-saving, noninvasive, quick and easy-to-use measure (18,43,47,48), it might be used to screen individuals with MetS in epidemiological studies (20). Because of the limitations of BF% assessment (overnight fast necessary, not applicable in women during the menstrual cycle or in individuals performing moderate to vigorous physical activity before measurement, and ethnic variation) (49), NC might be a more convenient option to detect MetS in adolescents. Compared to the other anthropometric measurements investigated in the present study, NC showed strong correlations with SBP and HDL-c, both MetS components. These results are consistent with other authors who also observed a strong relationship between SBP and NC in a study conducted in Lithuania on 1,947 adolescents aged 12-15 years (50).

Although BMI was the stronger predictor and showed higher accuracy in identifying MetS, the use of NC has advantages. For example, NC eliminates the need for a scale, stadiometer and undressing the subject, thus reducing the time necessary for evaluation and permitting to increase the number of subjects to be investigated (43). Given its good sensitivity and specificity, NC may be used for screening purposes, especially among people living in remote areas and in low-resource settings or when it is difficult to obtain weight and height (43). Furthermore, NC 1) is a proxy of upper BF distribution (47,48), which is strongly associated with the risk of CVDs and diabetes; 2) is not affected by postprandial abdominal distensions, avoiding false results (16,19,47); 3) is more acceptable among overweight and obese people (19,47), and 4) can be used both in research and in clinical settings to identify the risk of MetS in adolescents.

The use of sex- and age-specific criteria to identify adolescents with MetS was a strength of this study (6). Additionally, the use of a continuous variable (MetS score) instead of a categorical variable (MetS: yes or no) to detect MetS in adolescents has been recommended (12,36,41,42), especially due to the limitation of logistic regression in studies involving a small number of individuals with MetS (12). The prevalence of MetS in adolescents is low. In this respect, considering the outcome as a continuous variable increases the statistical power of the test (36,42) and avoids the loss of information that occurs when continuous variables are reclassified into categorical variables (41). For clinical purposes, since there are no accepted criteria for the definition of MetS in children and adolescents and because of the growing prevalence of MetS in this population, the use of a continuous variable to detect MetS seems to be an effective strategy to prevent the progression of MetS and associated pathologies in young people.

Another important strength of this study was the simultaneous association of multiple anthropometric measurements, including BMI, hip circumference, NC, triceps and subscapular skinfolds and BF%, with MetS in the same population of adolescents. This approach is important for clinical practice since it permits better comparison of different anthropometric indicators to diagnose MetS and to identify the best parameter to be used. Finally, this study has some limitations. First, its cross-sectional design does not allow conclusions about causality. Second, the lack of an international consensus classification of MetS in adolescents restricts the comparison of results between studies. Third, the small number of participants with MetS may limit the generalizability of our findings. Lastly, the MetS score derived in this study cannot be compared to other studies since it is sample specific.

In conclusion, all anthropometric measurements were associated with MetS score, with BMI showing the strongest relationship. However, in situations in which the measurement of height and weight is not possible, NC might be an interesting surrogate measurement because it can be obtained only with a tape measure at no cost. Health professionals should be made aware of this important tool for predicting MetS, even in apparently healthy adolescents. Since risk factors for MetS progress from childhood into adulthood, early lifestyle interventions are important to reverse the rising trend of noncommunicable diseases in adolescents. This approach may help decrease or prevent the onset of CVD and type 2 diabetes in adulthood, thus reducing the economic burden for the public health system.
